# Continuing medical education during a pandemic: an academic institution’s experience

**DOI:** 10.1136/postgradmedj-2020-137840

**Published:** 2020-05-13

**Authors:** Abhiram Kanneganti, Ching-Hui Sia, Balakrishnan Ashokka, Shirley Beng Suat Ooi

**Affiliations:** Department of Obstetrics and Gynaecology, National University Hospital, Singapore; Department of Cardiology, National University Heart Centre, Singapore; Department of Medicine, Yong Loo Lin School of Medicine, National University of Singapore, Singapore; Department of Anaesthesia, National University Hospital, Singapore; Centre for Medical Education, Yong Loo Lin School of Medicine, National University of Singapore, Singapore; Emergency Medicine Department, National University Hospital, Singapore; Department of Surgery, Yong Loo Lin School of Medicine, National University of Singapore, Singapore

**Keywords:** audit, telemedicine, education & training (see medical education & training), health policy, medical education & training

## Abstract

The COVID-19 pandemic has affected healthcare systems worldwide. The disruption to hospital routines has affected continuing medical education (CME) for specialty trainees (STs). We share our academic institution’s experience in mitigating the disruption on the CME programme amidst the pandemic. Most specialty training programmes had switched to videoconferencing to maintain teaching. Some programmes also utilized small group teachings with precautions and e-learning modules. Surgical residencies were disproportionately affected due to reductions in elective procedures but some ways to provide continued surgical exposure include going through archived surgical videos with technical pointers from experienced faculty and usage of surgical simulators . We should adapt CME sessions to keep trainees up to date with core clinical competencies as they will continue to manage both COVID-19 and non-COVID-19 cases and this pandemic may last until year’s end.

Since February 2020, COVID-19 has infected more than a million people and taken almost 50 000 lives with 50 000 cases and 5000 deaths in the UK alone. It has overwhelmed healthcare systems across the world with several countries instituting nationwide lockdowns, border controls and social distancing measures to curb spread. In the UK and several other countries, final year medical students are being fast-tracked to serve on the front lines.^1^ For Singapore, this is reminiscent of the severe acute respiratory syndrome (SARS) outbreak of 2003 where 238 were infected and 33 died. Then, decisive leadership minimised casualty count through a systematic process of hospital containment, rigorous surveillance and isolation, wide-spread community temperature screening, border controls, closure of schools and public engagement.^2^ A major strategy in ensuring continuity of healthcare services included segregating healthcare professionals (HCPs) into teams divided by time, place and skillset. Resultant increases in manpower and service needs coupled with the avoidance for congregation meant that all non-time-critical administrative and training functions ceased. Training for specialty trainees (STs) were casualties and were neglected for the 6 months the outbreak raged with medical education both in Singapore^3^ and globally^4–7^ suffered. Lessons were learned with three key understandings: (1) pandemics can be prolonged, (2) high-quality non-pandemic medical care is still a priority and (3) disruption to various functions can be reduced through technology.

Continuing medical education (CME) refers to structured, scheduled sessions directed toward all STs within a specialty training programme. They promote the upkeep of clinical skills and knowledge, maintain performance and ensure good patient outcomes^8^ and are thus crucial in enabling STs to continue delivering high-quality patient care. With this pandemic likely to last until this year’s end, we need to keep our STs equipped to deal with the continual non-COVID-19 cases and ready to face an ever-growing set of unmet clinical needs that are being postponed so we can deal with the current crisis. As our STs are on the front lines, it is important not to ignore their training. Just as various companies are innovating with work from home methods, it may be worthwhile to use technology to innovate and find ways to continue training.

The first COVID-19 case in Singapore was detected on 23 January 2020. By 7 February 2020, Singapore had 33 cases and escalated her pandemic alert to the penultimate level of Disease Outbreak Response System Condition (DORSCON)-Orange. Similar restrictive measures to the SARS outbreak^9^ were instituted with a return to HCP team segregation once again. Immediately, traditional didactic and small-group ST teachings were discontinued. The National University Hospital (NUH), Singapore is a tertiary academic medical institution and is host to 32 specialty training programmes (table 1). Several of our training programme directors and educational supervisors had experienced the SARS outbreak first-hand and were aware that this pandemic would be prolonged. Once the ground had settled, it was important to resume CME to equip our STs with the skills to manage both COVID-19 and non-COVID-19 cases. In the past decade, the development of user-friendly and accessible videoconferencing applications coupled with the widespread usage of smartphones and nationwide stability of 4G networks have made videoconferencing an effective option in transiting postgraduate medical education to virtual platforms.

**Table 1:** Thirty-two specialty training programmes at the National University Hospital, Singapore, as well as different methods taken to ensure the continuation of continuing medical education in each of them

Specialty training programmes		Methods used
**Medical**		
1.	Internal Medicine	Videoconferencing
2.	Endocrinology	
3.	Nephrology	
4.	Infectious Diseases	
5.	Respiratory and Critical Care	
6.	Neurology	
7.	Gastroenterology	
8.	Rheumatology	
9.	Medical Oncology	
10.	Haematology	
11.	Cardiology	
12.	Dermatology	
13.	Geriatrics	
14.	Paediatrics	
15.	Family Medicine	
16.	Preventive Medicine	Mobile-phone based regular updates
17.	Psychiatry	Videoconferencing Small-group didactics with adequate social distancing
**Surgical**		
18.	General Surgery	Videoconferencing Surgical videos Surgical simulators
19.	Orthopaedics	
20.	Hand Surgery	
21.	Cardiothoracic Surgery	
22.	Neurosurgery	
23.	Obstetrics and Gynaecology	
24.	Urology	
25.	Paediatric Surgery	
26.	Plastic Surgery	
27.	Otorhinolaryngology	
28.	Ophthalmology	
**Others**		
29.	Anaesthesia	Videoconferencing
30.	Pathology	Videoconferencing
31.	Radiology	Videoconferencing
32.	Emergency Medicine	Videoconferencing Small-group didactics with adequate social distancing Procedural videos

Of the 32 training programmes, 75% had to discontinue their CME programmes immediately once DORSCON-Orange was announced as they adjusted to this new working environment. Within the first 2 weeks, about 45% of training programmes had managed to restructure and resume sustained CME programmes. This increased to 70% by 4 weeks and 85% at the end of 8 weeks. Of the 27 training programmes that have successfully resumed their CME programmes, all have switched to videoconferencing for synchronous distance learning using either Zoom® (Zoom Video Communications, San Jose, California, USA) or in-house software. These sessions are delivered across a variety of mobile and computer platforms and presenters use voice-over, screen sharing and recording functions to deliver synchronous and asynchronous learning to STs on duty, at home or in commute. It also allows for interactive engagement and collaboration and can be applied to tumour boards, journal clubs and case-based discussions. Presenters can use interactive web-based audience response systems for preteaching and post-teaching evaluation and assessment through platforms such as Google Forms (Alphabet, Mountain View, California, USA). The remaining five training programmes that have not yet resumed regular CME sessions are small and resultantly have a very high degree of interaction between the STs and their educational supervisors. They are taking active steps toward creating specialty-specific CME programmes which will come online in the next few weeks. Nevertheless, increased service needs and dyssynchronous rosters have caused 35% of training programmes to continue CME sessions at a reduced frequency to cope in this new operating environment. This highlights that even though videoconferencing can be used to continue CME, it would understandably have to compete and coexist with the significantly higher clinical priorities of the day that is, this pandemic.

Respiratory Medicine, Preventive Medicine, Emergency Medicine and Infectious Diseases STs are deeply involved in this pandemic. Respiratory Medicine had been able to restart their CME programme within 2 weeks, while Emergency Medicine and Infectious Diseases took about 6 weeks as the rapidly evolving COVID-19 situation necessitated an urgent need to develop protocols and redistribute manpower. Preventive Medicine STs were dispersed early on to various bodies to assist with pandemic management and so they replaced their formal CME programme to one which was more mobile phone-based and tailored to sharing the latest updates, literature and guidelines on COVID-19.

Other disciplines have taken different approaches. Psychiatry continue to have small-group teachings among two to three STs with appropriate physical distancing in addition to videoconferencing, which is appropriate given their small ST cohort size and a need for in-person mental state examinations. Disciplines dealing with oncology such as Medical Oncology, Pathology, Radiology and surgical specialties traditionally included multidisciplinary tumour board sessions as part of their CME. Tumour boards had already been using videoconferencing prepandemic and oncological work needed to be continued given its time-critical nature. As a result, CMEs through tumour boards continued without disruption and transited effortlessly into a fully virtual environment.

Non-oncological elective surgical work has been reduced by 80% to reserve surge capacity for critical care and other resources. As a result, surgical training programmes have been disproportionately affected by a reduction of hands-on training opportunities. Usage of in-house surgical simulators are being reviewed to address this shortfall. Videoconferencing has been successfully used in surgical education^10^ and Obstetrics and Gynaecology uses recorded surgical videos to go through anatomical and surgical principles.

Several colleges have also postponed important milestone examination.^11^ This is a source of great distress to any ST as many life decisions are frequently put on hold and significant effort goes into staying prepared. While multiple-choice questions can be practised with question books, Objective Structured Clinical Examination (OSCE) require rehearsal with live participants. OSCEs involving simulated patient encounters and topic discussion have been conducted over videoconferencing successfully. OSCEs requiring clinical signs, however, may need some creativity to be simulated. Various colleges also publish a rich variety of e-learning modules which are examination oriented and should be explored fully as form of asynchronous learning.

This review of NUH’s teaching practice shows that innovation, with the use of technology, can adapt specialty training programmes in pandemics, although with modification and reduced frequency. While CME was initially disrupted as various training programmes adapted to a new segregated operating environment and developed new protocols, it was restarted by most programmes by the fourth week. These virtual CME sessions have additional benefits of serving as a two-way forum for feedback from STs on the ground and for topical updates on COVID-19 literature and protocols. STs will face increased stress and fatigue as a result of increased workload and emotional toll during this pandemic.^12^ Several STs have stated that regular virtual CME sessions serve as a means of peer support and solidarity at a time of isolation and segregation. This likely helps with individual coping mechanisms.

This pandemic is projected to last until the end of the year^13^. We should adapt CME to a ‘new normal’ to keep our STs up to date with their core clinical competencies and equipped to deal with the full spectrum of clinical pathology that will no doubt continue now and tomorrow.

**List of learning points**

- The COVID-19 pandemic has caused disruption to continuing medical education. Specialty trainees will continue to be at the front lines looking after both COVID-19 and non-COVID-19 cases and they should be supported with training.
- Specialty trainees should, and can, continue to receive training with the use of distance learning technology such as videoconferencing, archived videos, simulators and e-learning modules.
- This pandemic may last until year’s end and Continuing medical education should be adapted to the 'new normal' to keep specialty trainees up to date.
